# Influence of Different Packaging Materials on the Composition of the Headspace of Rennet Cheeses under Different Modified Atmosphere Conditions

**DOI:** 10.3390/foods13162500

**Published:** 2024-08-09

**Authors:** Justyna Zulewska, Adriana Lobacz, Ireneusz Bialobrzewski, Adam Grochowina, Anna Kaminska

**Affiliations:** 1 Department of Dairy Science and Quality Management, Faculty of Food Science, University of Warmia and Mazury in Olsztyn, 10-719 Olsztyn, Poland; adriana.lobacz@uwm.edu.pl; 2 Department of Systems Engineering, Faculty of Technical Sciences, University of Warmia and Mazury in Olsztyn, 10-719 Olsztyn, Poland; irekb@uwm.edu.pl; 3 Hochland Polska Sp. z o. o., 07-100 Węgrów, Poland; adam.grochowina@hochland.com (A.G.); anna.kaminska@hochland.com (A.K.)

**Keywords:** foil packaging, cheese headspace, MAP

## Abstract

The aim of this study was to analyze the influence of different packaging materials on the composition of the headspace (CO_2_ and O_2_) of rennet cheeses packed in unit packaging under different modified atmosphere (MAP) conditions during a storage period of 90 days at 2 °C and 8 °C. The packaging materials comprised different combinations of BOPP—biaxially oriented polypropylene; PET—polyester; PE—polyethylene; PP—polypropylene; EVOH—ethylene–vinyl alcohol copolymer; PET—polyethylene terephthalate; and PA—polyamide. As the properties of the packaging material (foil) affect the gas conditions inside the packaging, it is important to study whether the modifications, i.e., properties and thickness, of the foils will result in significant differences in the composition of the headspace of packed cheeses. The CO_2_ content in the headspace of Gouda cheese packages ranged from 35% to 45%, while for Maasdamer and Sielski Klasyczny cheese, it varied between 55% and 65%. Throughout the storage period, the O_2_ content in the headspace of cheeses packaged in tested foils (1–5) did not exceed 0.5%. The type of foil used did not influence the modified atmosphere packaging (MAP) conditions.

## 1. Introduction

Recent amendments to legal regulations in the European Union concerning plastic usage, specifically Directive (EU) 2019/904, Directive (EU) 2018/852, and the European Commission’s Green Deal (2022), have led to an increase in environmentally sustainable practices among food manufacturers [1]. Any innovation utilizing thinner packaging must be appropriately designed to minimize CO_2_ loss, as this could potentially reduce the product’s shelf life [2,3,4].

Attempts were made to study the active modified atmosphere packaging of cheese using N_2_ and CO_2_ in the absence of O_2_ [5,6,7,8]. It is essential to select specific MAP conditions tailored to the material of the packaging and the type of product being packaged.

Modified atmosphere packaging (MAP) has become extensively employed by both large and small food producers to mitigate issues related to oxygen-sensitive foods [9]. The MAP technique employs gas mixtures with reduced oxygen levels to extend the shelf life of hard cheeses by providing protection against oxidation and inhibiting the proliferation of spoilage microorganisms [10]. Consequently, hard, and semi-hard cheeses are typically enclosed in laminate combinations of polyamide and polyethylene and packaged in 100% carbon dioxide or mixtures of carbon dioxide and nitrogen using horizontal form–fill–seal pouch packing equipment [10,11].

Despite the extensive utilization and automation of the MAP process, it is not consistently 100% effective in achieving the desired gas composition. Consequently, the presence of minor amounts of residual oxygen, ranging from 0.0% to 2%, has been reported in packages packed under an oxygen-free atmosphere [12]. Elevated levels of residual oxygen in all MAP packages can stem from the gas permeability of the packaging material and the food’s capacity to retain air. Furthermore, some packages may experience ineffective gas flushing, compromised thermoforming or sealing processes, or incidental minor damage during handling or transportation, leading to significant air ingress into the packages [13]. These factors can compromise the quality and safety of products throughout storage, distribution, and consumption, leading to issues such as fat oxidation, microbiological contamination, and loss of moisture and aroma [14].

The gas composition within a package at equilibrium is determined by the interactions among the product, barrier material, and environmental conditions [15]. Consequently, the design of a modified atmosphere package is contingent upon numerous variables associated with each of these factors. Regarding the product to be packaged, the considerations include the gas exchange rate influenced by O_2_, CO_2_, and temperature, product weight, and recommended atmosphere [14]. Concerning the barrier material, if the packaging system comprises a polymeric flexible film, factors such as the gas permeability, surface area, and thickness are pertinent; however, if the packaging system involves an impermeable covering with perforations, considerations encompass the dimensions and shape of the covering, number of perforations, and their area and length. Finally, environmental factors influencing MAP design encompass temperature, relative humidity, and concentrations of O_2_ and CO_2_ [16].

The aim of this study was to analyze the influence of different packaging materials (standard foil: BOPP/PET/PE for upper layer and APET/PET for bottom layer; tested foil 1: PP/PET/PE/EVOH/PE and PP/PE/EVOH/PE, tested foil 2: PP/PET/PE/EVOH/PE and PA/EVOH/PE, tested foil 3: PP/PET/PE and PA/EVOH/PE, tested foil 4: PP/PET/PE and PA/PE, tested foil 5: PP and PP/PP) on the composition of the headspace (CO_2_ and O_2_) of rennet cheeses packed in unit packaging under different modified atmosphere (MAP) conditions during period of 90 days of storage at 2 °C or 8 °C.

## 2. Materials and Methods

### 2.1. Experimental Design

Three types of sliced cheeses—Gouda, Maasdamer, and Sielski Klasyczny (Hochland Sp. z o.o., Węgrów, Poland)—were individually placed in six distinct packaging materials, consisting of one standard (control sample) and five tested foils that were subjected to analyses (Table 1).

The standard packaging involved the application of a modified atmosphere (CO_2_: N_2_) with specific ratios (Gouda: 40:60; Maasdamer and Sielski Klasyczny: 60:40). Each of the tested packaging materials underwent three different conditions (Gouda: 35:65; 40:60; 45:55; Maasdamer and Sielski Klasyczny: 55:45; 60:40; 65:35). Cheese samples were evaluated after 7, 30, 60, and 90 days from packaging, and various storage temperatures were implemented. Upon receipt, individual cheese packages were promptly refrigerated and stored at 2 and 8 °C (within the refrigerator temperature limits). The design of experiment involving packaging material type, modified atmosphere packaging (MAP), temperature, and sampling day are presented in Table 2.

### 2.2. Analyses of the Gas Composition

The gas composition (O_2_ and CO_2_ concentrations) of the headspace of all packages was determined prior to opening by using a CheckMate3 (PBI Dansensor, Ringsted, Denmark). The changes in gas composition inside each package were monitored over time (7, 30, 60 and 90 days of storage) by taking samples through a septum of the package with the help of a 0.8 mm needle attached to a gas analyzer equipped with an electrochemical sensor for gas measurement with an accuracy of ±0.1 and ±0.5 absolute for O_2_ and CO_2_, respectively. The content (%) of O_2_ and CO_2_ in the package atmosphere was determined, while the content of N_2_ was calculated as 100 minus the content of O_2_ (%) and CO_2_ (%).

### 2.3. Instrumental and Chemical Analyses

The cheese samples were grated (Santos type 02 cheese grater, Lyon, France) for all analytical purposes. The protein content in the cheese samples was determined using the Kjeldahl method (AOAC, 2007; method 2001.14, 33.7.12 A [18]) by calculating the total nitrogen and multiplying by 6.38. Fat content was determined via the Schmid–Bondzyński–Ratzlaff method (ISO, 2004; ISO 1735:2004, IDF 5:2004 [19]). The moisture content was assessed gravimetrically by drying 2 g of cheese in a forced-air oven (FD 53, Binder, Tuttlingen, Germany) at 102 °C for 24 h (AOAC, 2007; 33.2.44, 990.20 [20]). The pH of the cheese was measured using a Beckman ϕ 72 pH meter (Beckman Instruments Inc., Indianapolis, IN, USA) equipped with an InLab SolidsPro pH electrode (Mettler Toledo, Columbus, OH, USA). The salt content was determined using a FoodScan analyzer (Foss, Hillerod, Denmark).

### 2.4. Statistical Analysis

To assess the significance of the effect of time on the measured quantities, an analysis of variance with repeated measurements was used for the experimental systems, which could be defined as systems with repeated measurements. For all other experimental systems, multivariate analysis of variance was used to determine the effects of three factors (film, MAP, time) and two factors (film, MAP) on the headspace composition (CO_2_ and O_2_) of rennet cheeses, including their interactions. Statistical analyses were performed at a significance level of *p* = 0.05, using the statistical procedures ranova and anovan, which are available in the Statistics and Machine Learning Toolbox of the Matlab 2020a package (MathWorks, Natick, MA, USA).

## 3. Results

### 3.1. Composition

The composition of the cheeses was typical for specific cheese categories. The Gouda cheese was full-fat type cheese, whereas Maasdamer and Sielski Klasyczny were medium-fat cheeses according to the Codex General Standard for Cheese [21]. Small but significant (*p* < 0.05) changes were detected for most of the analyzed components for all studied cheeses stored at 2 °C over the period of 90 days (Table 3).

**Figure 1:**
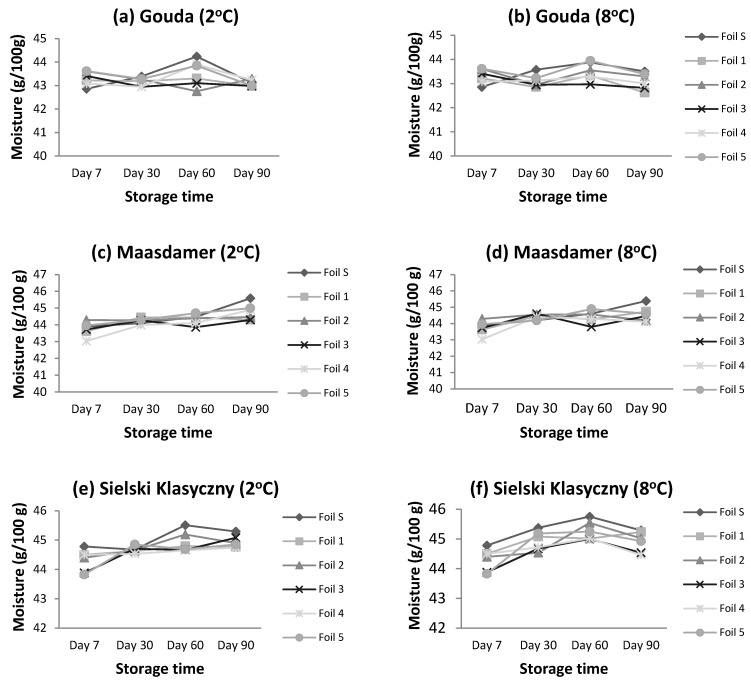
Changes in moisture content for studied cheeses stored at 2 and 8 °C. Means (n = 3) were calculated for standard and tested foils in different modified atmosphere of packaging conditions.

The moisture content did not change for Gouda cheeses stored at 2 °C for the period of 3 months, and it tended to increase in case of Massdamer and Sielski Klasyczny cheese. For Gouda and Maasdamer cheeses stored at 8 °C for 3 months, no change in moisture levels was noted. On the other hand, for Sielski Klasyczny cheese at 8 °C storage temperature, there was an increase in moisture until the 60th day of storage to an average level of 45.3 g/100 g, after which there was a decrease in moisture content to 44.9 g/100 g. The changes in the moisture content of the three studied cheeses stored at 2 and 8 °C are presented in Figure 1.

**Figure 2:**
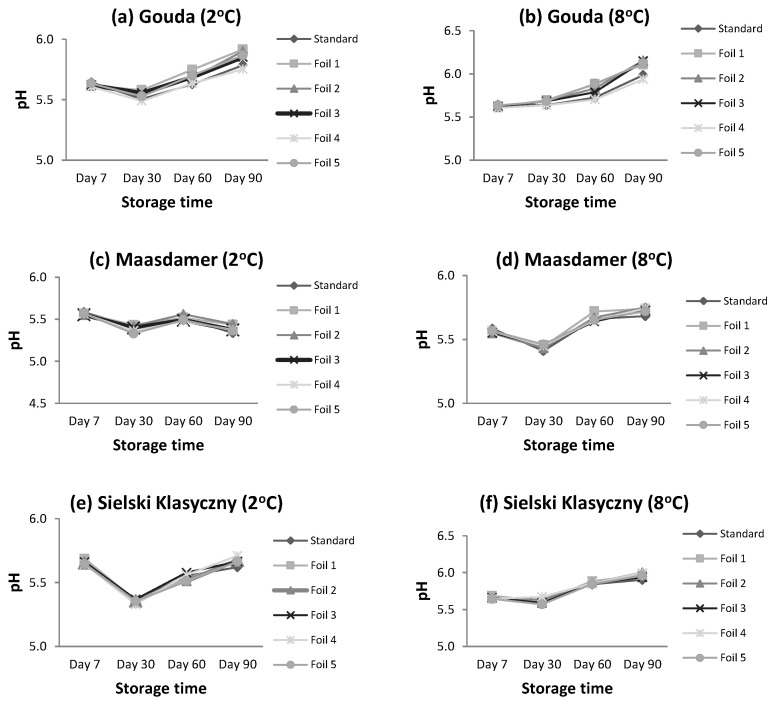
pH changes in moisture content for studied cheeses stored at 2 and 8 °C. Means (n = 3) were calculated for standard and tested foils in different modified atmosphere of packaging conditions.

For most of the studied cheeses, there was a slight increase in pH value during storage (Table 3). The only exception was Maasdamer cheese stored at 2 °C, for which the pH decreased (*p* < 0.05). At 8 °C, the increase in pH was higher. The changes in pH value were the lowest for Maasdamer cheese. In turn, Gouda cheese showed the biggest shift in pH during storage for each temperature variant. The pH changes of the three studied cheeses stored at 2 and 8 °C are presented in Figure 2.

### 3.2. Gas Composition

**Figure 3:**
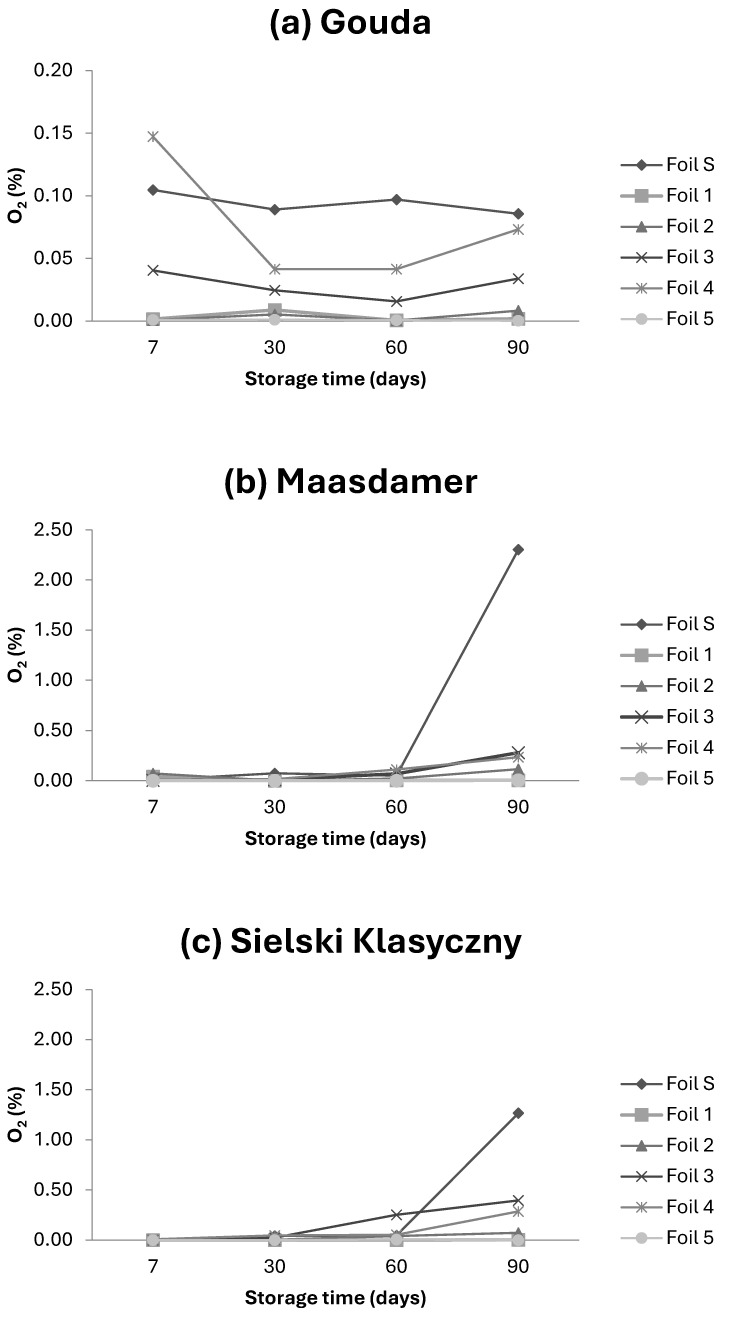
Changes in oxygen (O_2_) content for Gouda (**a**), Maasdamer (**b**) and Sielski Klasyczny (**c**) cheese packed in different foils during 90-day storage. The mean (n = 3) was calculated for samples stored at 2 °C with different MAP conditions.

Oxygen is directly or indirectly implicated in the primary instances of spoilage observed in hard cheeses [10]. The oxygen content for most of the studied samples was at a very low level (Figure 3).

The initial (day 7) content of oxygen in the packages was below 0.1% with the exception of tested foil no. 4 for which the mean value of 0.147% was calculated. In general, the Gouda cheese did not show any significant changes in oxygen content during 90-day storage at 2 °C. The packages of Maasdamer and Sielski Klasyczny showed slight increases in O_2_ content during storage at 2 °C; however, for the tested foils (foils 1–5), the content of O_2_ after 90 days was still below 0.4%. The cheeses Maasdamer and Sielski Klasyczny, packed in standard foil, showed higher O_2_ content after 90 days of storage, reaching the values of 2.3 and 1.3%, respectively. The O_2_ transmission rate (cm^3^/m^2^ 24 hxbar) varied between the foils with foils 3 and 4 having the highest range (110–130) and foils 1, 2 and 5 having the lowest (Table 1) for the upper layer. However, it is worth noting that for cheeses packed in thinner foils (3–5), regardless the temperature of storage, the oxygen did not exceed 0.5%.

**Figure 4:**
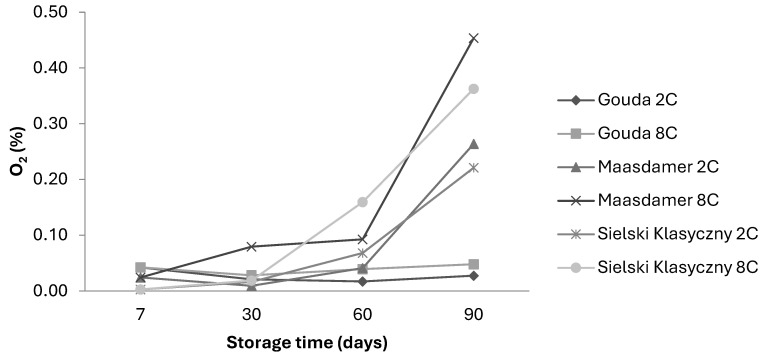
Changes in oxygen (O_2_) content for Gouda, Maasdamer, and Sielski Klasyczny cheese stored at 2 and 8 °C. The means (n = 15) were calculated for tested foils (foil 1–5) in different MAP conditions.

Considering the effect of storage temperature on the oxygen content in the packages, the change in O_2_ content was more noticeable when the cheeses were stored at higher temperatures of 8 °C (Figure 4).

In general, the time and type of foil had a significant effect on the oxygen content in the headspace of the packages for cheeses stored at 2 °C, while at 8 °C, time was the most important variable affecting O_2_ content only for Massdamer and Sielski Klasyczny. For Gouda cheese stored at 8 °C, foil type and MAP conditions explained more variations over storage time (Table 4). In general, there was no effect of MAP conditions on the function of time on CO_2_ content for Maasdamer and Sielski Klasyczny cheeses; however, Gouda cheese showed a significant effect (Table 4). Most probably, the type of the starter cultures used during the production of the given cheese and their ability to produce CO_2_ may also contribute to changes in the composition of the headspace of the package.

**Figure 5:**
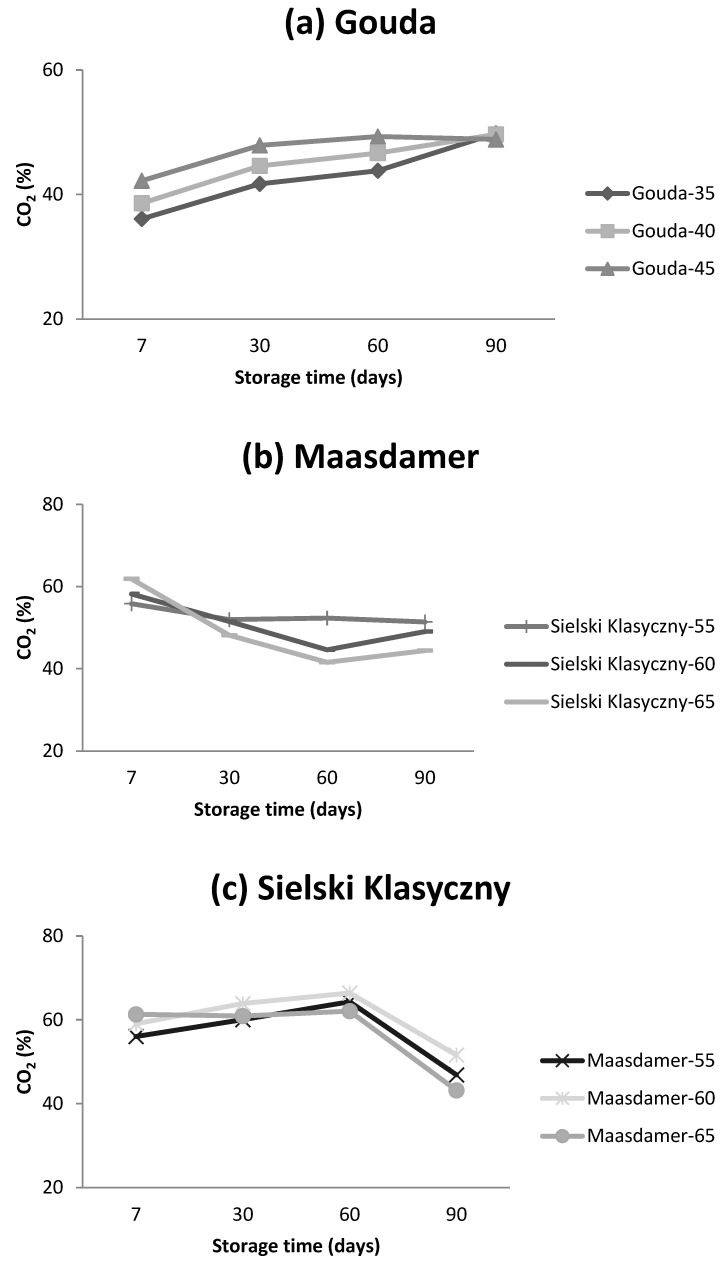
Changes in CO_2_ content for (**a**) Gouda, (**b**) Maasdamer, and (**c**) Sielski Klasyczny cheese depending on the starting level of CO_2_. The mean (n = 5) was calculated for samples packed in tested foils (foil 1–5) stored at 2 °C.

The changes in CO_2_ content in cheese packages were detected as a function of storage time (Figure 5a–c, Table 5). It can be concluded that for a particular cheese category, there was a similar pattern in the changes of CO_2_ during storage (90 days) (Figure 5a–c).

**Figure 6:**
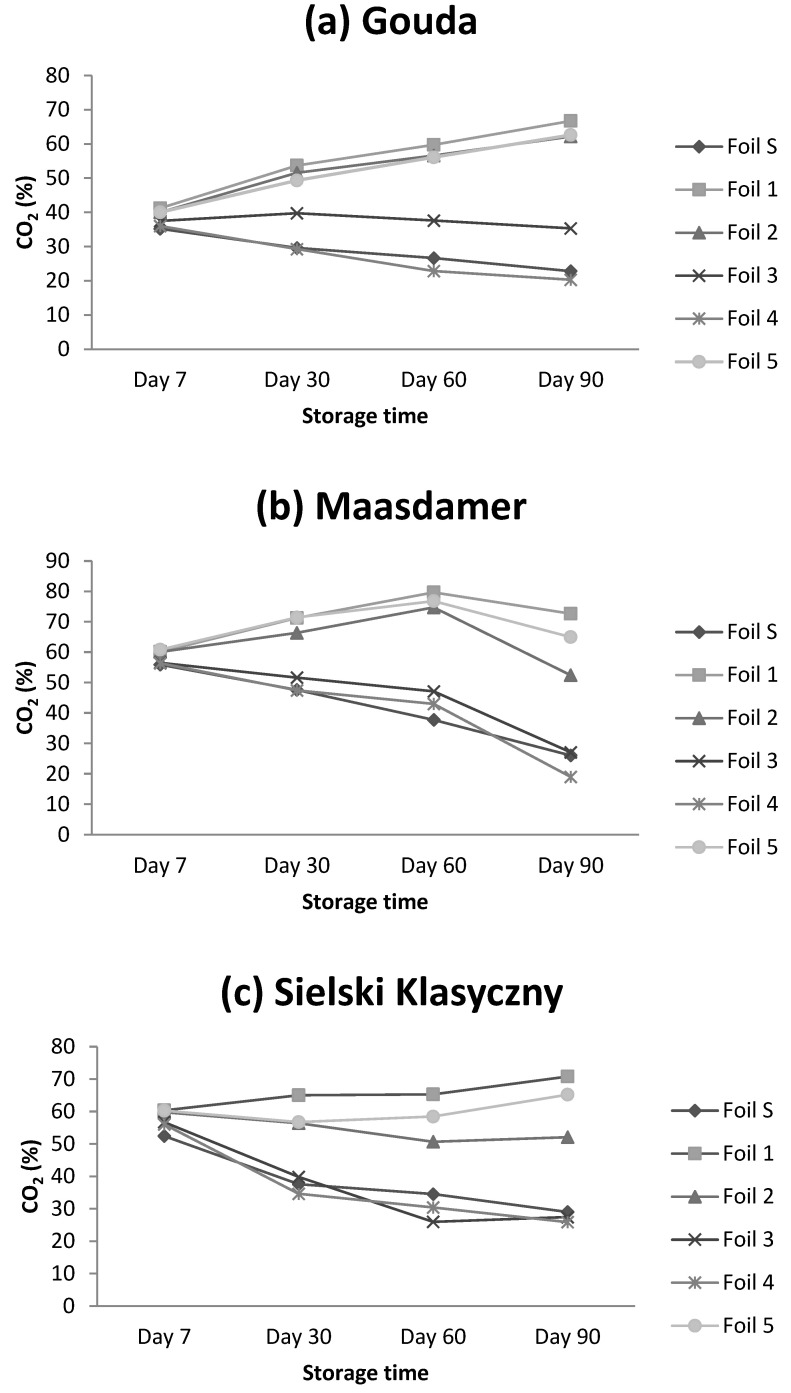
Changes in CO_2_ content for Gouda (**a**), Maasdamer (**b**), and Sielski Klasyczny (**c**) cheese depending on the type of packaging material. The mean (n = 3) was calculated for samples stored at 2 °C under different MAP conditions.

For Gouda cheese, the CO_2_ content in the headspace of the packages increased over the storage period. This increase was inversely related to the initial CO_2_ levels with lower initial percentages resulting in a more pronounced increase. Conversely, Maasdamer and Sielski Klasyczny cheeses exhibited a decrease in CO_2_ content after a 90-day storage period. For these cheeses, higher initial CO_2_ levels corresponded to a greater decrease by the end of the storage period (Figure 5). Notably, the changes in CO_2_ content within the package headspace were dependent on the type of packaging foil used (Table 5). In case of all cheeses, the CO_2_ content in packages made of standard foil decreased during the storage period (Figure 6). For Gouda cheese packed in tested foils 1, 2 and 5, the CO_2_ level increased during storage. The changes in CO_2_ content in case of foil 3 and 4 showed the same pattern as in case of standard foil. Similar clustering was observed for Maasdamer and Sielski Klaasyczny cheeses. In case of Maasdamer and Sielski Klasyczny cheese, the CO_2_ in the headspace of the package made of foil 1 and foil 5 showed an increase; however, the increase in case of foil 5 was less pronounced. For the foils 2, 3 and 4, for both cheeses, the CO_2_ level tended to decrease after 90 days. However, the CO_2_ content in the packages of Maasdamer cheese showed an initial increase (up to 60 days of storage) and then decreased. For Maasdamer and Sielski Klasyczny, the decrease in CO_2_ content was more pronounced for cheeses packed in foils 3 and 4 (Figure 6).

## 4. Discussion

Modified atmosphere packaging (MAP) is a well-established technique that involves altering the gas composition surrounding a product, creating an atmosphere distinct from that of air. The optimal atmospheric composition for each product is achieved by aligning the permeability of the packaging material with the specific product’s rates of CO_2_ production and O_2_ consumption [22]. In addition to the composition of the modified atmosphere, the selection of appropriate packaging material is a crucial factor influencing the selected quality characteristics of cheese. Scientific reports indicate that the properties of barrier foils, such as thickness, significantly affect the water content and hardness of tvarog cheese (Polish acid-coagulated white, fresh cheese) stored under refrigerated conditions. However, for PE/EVOH/PA foils of various thicknesses, no clear influence on the fat content, titratable acidity, pH, or sensory properties of the experimental tvarog cheese was observed [23]. Pluta et al. (2013) investigated the impact of modified atmosphere packaging (MAP) with varying gas compositions and packaging material thicknesses on the quality characteristics of sliced Swiss cheese [24]. Their findings indicated no correlation between the type of foil used and the microbiological quality of the product. Currently, the literature lacks definitive evidence to establish a relationship between specific packaging materials and the quality characteristics of various cheese types. Thus, it is essential to conduct further studies on suggested packaging materials to elucidate the relationship between cheese packaging methods and the quality of the final product.

Oxygen facilitates several types of deteriorative reactions in foods, including fat oxidation, browning reactions, and pigment oxidation. Additionally, most common spoilage bacteria and fungi require O_2_ for growth. Consequently, to extend the shelf life of foods, the packaging atmosphere should contain a low concentration of residual O_2_ [25]. In general, the level of CO_2_ was the highest for cheeses packed in tested foils 1, 2 and 5. The reason for that could be the composition of the foils used. Foils 1 and 2 (Table 1) were comprised of EVOH (both lid and bottom), which is characterized by high barrier features (many times exceeding the characteristics of typical, massively used plastics), an excellent oxygen barrier, high transparency, a flavor barrier, high rigidity and easier processability [26]. Tested foil 5 consisted of PP, which in fact has a good water vapor barrier but a poor gas barrier [25]; however, an additional layer of barrier was applied for this foil. In the present study, among the tested foils (1–5), the thinnest ones were foil 1 (lid: 10 µm and bottom: 93 µm) and foil 5 (lid: 65 µm and bottom: 85 µm), which were characterized also by the lowest O_2_ transmission rate (Table 1). Polypropylene (PP) is semi-rigid, translucent, and possesses good chemical resistance, toughness, fatigue resistance, and heat resistance as well as an integral hinge property, while polyester (PET) offers good chemical resistance and does not react with foods and liquids. Polyethylene (PE) is semi-rigid, translucent, very tough, weatherproof, and characterized by good chemical resistance, low water absorption, ease of processing, and low cost; polyamides, used in the bottom foil, are favored for their mechanical strength, high heat distortion temperature, flexibility, toughness, and excellent barrier properties against oxygen, chemicals, and aromas as well as their high transparency and thermoformability [1].

The data reported in the literature [27,28,29,30] clearly demonstrate the potential of modified atmosphere packaging (MAP) for extending the shelf life of dairy products, including cheese. Success in cheese packaging depends on several critical parameters, including the type of cheese, the use of starter cultures during production, initial microbial contamination levels, and storage conditions. It is recommended to apply a high % N_2_ for semi-hard or hard type cheeses packaging [8,31].

Moreover, the type of applied cultures affects the rate and amount of CO_2_ production [32]. In the case of a cheese block weighing ca. 80 kg, the production of carbon dioxide during ripening is about 120 liters. Half of this amount is dissolved in cheese, approx. 20 liters remain in the eyes and approx. 40 liters diffuse out of the cheese block [33]. As nearly as one third of the CO_2_ produced during ripening leaves the cheese matrix, another technological problem is the influence of the additives used for cheese manufacture, and particularly the influence of the used starter cultures, on the atmosphere composition in the packages. According to the literature, the differences in the content of CO_2_ and O_2_ in the packaging may be caused by the cheese respiration during ripening and storage, oxidation processes, and low oxygen permeability through the packaging material [31]. Therefore, it is reasonable to analyze the CO_2_ and O_2_ content in the packages depending on the storage time of the cheese. According to Juric et al. (2003), the most significant changes in the gas composition—an increase in CO_2_ content—are expected to occur within two days of storage [31]. The observations concern 5–6 weeks-old semi-hard cheeses sliced, and packed in a modified atmosphere. In our case, propionic bacteria were used in the production of Maasdamer and Sielski Klasyczny cheese.

In general, rigid materials of small thickness possess relatively weak gas barrier properties. The thickness that is necessary to achieve acceptable rigidity increases the properties of the gas barrier to a level suitable for many applications. Adding another barrier, e.g., EVOH, will extend the shelf life of the packed product. The choice of films for MAP depends largely on their gas and water vapor permeability. Materials such as polyester (PET), nylon (PA), polyvinylidene chloride (PVdC) and ethylene–vinyl alcohol copolymer (EVOH) provide good barrier to gases, but in many cases, they provide a poor barrier to water vapor. PP and PE have a good barrier for water vapor and aromas, but it is low for gas penetration [25].

The storage temperature is also a very important factor influencing the product behavior with higher temperatures promoting the changes. Moreover, the storage temperature may affect the properties of the packaging material, i.e., O_2_ or CO_2_ transmission rate, thus affecting the composition of the gases inside the package. Different polymers, such as polyethylene (PE), polypropylene (PP), and polyamide (PA), are widely used in the packaging of food; however, their use depends on the properties of the final product [34]. The procedures applied during manufacturing, handling and packaging can impact the barrier properties of flexible packaging materials [34].

## 5. Conclusions

The O_2_ content of the headspace of the cheeses packaged in experimental foils (1–5) did not exceed 0.5% during the storage period. The foil type had no significant effect on MAP conditions. It was possible to thicken the packaging material from the initial 103/250 µm (standard foil), for the lid and bottom, respectively, to 98/100 µm (tested foil 4) without affecting the attributes of the rennet cheeses.

## Figures and Tables

**Table 1:** Composition of packaging materials: lid and bottom foils.

	Lid Foil			Bottom Foil		
Foil	Composition	Thickness (µm)	O_2_ Transmission Rate ^1^ (cm^3^/m^2^ 24 hxbar)	Composition	Thickness (µm)	O_2_ Transmission Rate (cm^3^/m^2^ 24 hxbar)
Standard	BOPP ^2^/PET ^3^/PE ^4^	103	101.3	PET ^7^/PE	250	17
Tested foil 1	PP ^5^/PET/PE/EVOH ^6^/PE	10	1–3	PP/PE/EVOH/PE	93	1–3
Tested foil 2	PP/PET/PE/EVOH/PE	101	1–3	PA ^8^/EVOH/PE	120	<3
Tested foil 3	PP/PET/PE	98	110–130	PA/EVOH/PE	120	<3
Tested foil 4	PP/PET/PE	98	110–130	PA/PE	100	<39
Tested foil 5	PP with barrier	65	<2	PP/PP with barrier	85	<2

^1^ O_2_ transmission rate: according to the standard ASTM D3985 [17]; ^2^ BOPP—biaxially oriented polypropylene; ^3^ PET—polyester; ^4^ PE—polyethylene; ^5^ PP—polypropylene; ^6^ EVOH—ethylene-vinyl alcohol copolymer; ^7^ PET—polyethylene terephthalate; ^8^ PA—polyamide.

**Table 2:** Design of experiment involving packaging material type, modified atmosphere packaging (MAP), temperature, and sampling day.

Foil	MAP		Temperature (°C)	Sampling Day
	Gouda	Maasdamer, Sielski Klasyczny		
Standard	40% CO_2_	60% CO_2_	2	7, 30, 60, 90
			8	30, 60, 90
Tested foil 1 Tested foil 2 Tested foil 3 Tested foil 4 Tested foil 5	35% CO_2_	55% CO_2_	2	7, 30, 60, 90
			8	30, 60, 90
	40% CO_2_	60% CO_2_	2	7, 30, 60, 90
			8	30, 60, 90
	45% CO_2_	65% CO_2_	2	7, 30, 60, 90
			8	30, 60, 90

**Table 3:** Mean (n = 15) composition (% by weight) and pH of the cheeses after 7, 30, 60, and 90 days from packing for the samples stored at 2 °C in tested foils (foil 1–5) with different MAP conditions.

Composition	Cheese Type											
	Gouda				Maasdamer				Sielski Klasyczny			
	7	30	60	90	7	30	60	90	7	30	60	90
Moisture (%)	43.39 ^a^ ± 0.31	43.14 ^a^ ± 0.42	43.38 ^a^ ± 0.58	43.09 ^a^ ± 0.26	43.73 ^b^ ± 0.77	44.26 ^a^ ± 0.40	44.30 ^a^ ± 0.41	44.59 ^a^ ± 0.61	44.22 ^b^ ± 0.38	44.67 ^a^ ± 0.33	44.80 ^a^ ± 0.61	44.87 ^a^ ± 0.31
Protein (%)	23.49 ^a^ ± 0.13	23.42 ^ab^ ± 0.15	23.36 ^bc^ ± 0.21	23.26 ^c^ ± 0.12	24.96 ^ab^ ± 0.33	25.08 ^a^ ± 0.26	25.19 ^a^ ± 0.16	24.79 ^b^ ± 0.22	24.97 ^a^ ± 0.23	24.92 ^a^ ± 0.23	24.88 ^a^ ± 0.20	24.90 ^a^ ± 0.29
Fat (%)	26.60 ^a^ ± 0.28	26.37 ^b^ ± 0.33	26.02 ^c^ ± 0.27	26.18 ^bc^ ± 0.14	24.27 ^a^ ± 0.42	24.30 ^a^ ± 0.21	23.62 ^b^ ± 0.31	23.60 ^b^ ± 0.27	24.78 ^a^ ± 0.33	24.08 ^b^ ± 0.25	23.67 ^c^ ± 0.25	23.61 ^c^ ± 0.26
Salt (%)	1.92 ^b^ ± 0.12	1.86 ^b^ ± 0.15	1.99 ^ab^ ± 0.12	2.04 ^a^ ± 0.07	1.73 ^a^ ± 0.11	1.66 ^a^ ± 0.11	1.69 ^a^ ± 0.15	1.75 ^a^ ± 0.06	1.47 ^b^ ± 0.10	1.53 ^b^ ± 0.18	1.58 ^ab^ ± 0.10	1.66 ^a^ ± 0.10
pH	5.62 ^bc^ ± 0.02	5.54 ^c^ ± 0.04	5.69 ^ab^ ± 0.05	5.86 ^a^ ± 0.09	5.56 ^a^ ± 0.02	5.39 ^b^ ± 0.05	5.51 ^a^ ± 0.07	5.40 ^b^ ± 0.04	5.66 ^a^ ± 0.03	5.35 ^c^ ± 0.03	5.54 ^b^ ± 0.08	5.68 ^a^ ± 0.03

^a–c^ means within a row for each cheese sharing a different superscript are different (*p* < 0.05).

**Table 4:** A three-way analysis of variance for the oxygen (O_2_) and carbon dioxide (CO_2_) content in the headspace of cheeses, packed in unit packaging and stored at both 2 °C and 8 °C, over a 90-day period using tested foils.

Parameter		Gouda			Maasdamer			Sielski Klasyczny		
		Source of Variation								
		Time	Foil Type × Time	MAP Conditions × Time	Time	Foil Type × Time	MAP Conditions × Time	Time	Foil Type × Time	MAP Conditions × Time
		Storage temperature 2 °C								
	df	3	12	6	3	12	6	3	12	6
O_2_	SS	0.0060 **	0.0177 **	0.0009	0.1318 **	0.1473 *	0.0232	0.2064 **	0.2723 **	0.0654
CO_2_	SS	880.50 **	2285.21 **	91.51 **	2533.84 **	3104.24 **	182.84	1339.17 **	2527.36 **	417.95
		Storage temperature 8 °C								
O_2_	SS	0.0019	0.0142 *	0.0083 *	1.1678 *	2.6831	0.5864	1.2575 **	1.2286	0.6224
CO_2_	SS	552.28 **	1690.36 **	248.95 **	9114.87 **	3078.50 **	223.90	8908.02 **	3357.24 **	211.55

df—degrees of freedom; SS—sum of squares; * *p* ≤ 0.05; ** *p* ≤ 0.01.

**Table 5:** A two-way analysis of variance for oxygen (O_2)_ and carbon dioxide (CO_2_) content in the headspace of cheeses, packed in unit packaging and stored at 2 °C and 8 °C, over a 90-day period using tested foils.

Parameter		Gouda			Maasdamer			Sielski Klasyczny		
		Source of Variation								
		Foil	MAP Conditions	Foil × MAP Conditions	Foil	MAP Conditions	Foil × MAP Conditions	Foil	MAP Conditions	Foil × MAP Conditions
		Storage temperature 2 °C								
	df	4	2	8	4	2	8	4	2	8
O_2_	SS	0.0344 **	0.0010	0.0012	0.5844 **	0.1131	0.4177 *	1.1621 **	0.2287 **	0.3433
CO_2_	SS	15136.1 **	8.2	119.5 *	19707.3 **	538.5 **	4005.5 **	15685.9 **	377.9 **	2991.4 **
		Storage temperature 8 °C								
O_2_	SS	0.0385	0.0214	0.0407	6.0799 **	0.9144	0.8875	4.9429 *	2.1633	4.2621
CO_2_	SS	9484.1 **	371.68 **	282.39	9848.14 **	18.01	1311.05 **	14642.31 **	38.35	1731.86 **

df—degrees of freedom; SS—sum of squares; * *p* ≤ 0.05; ** *p* ≤ 0.01.

## Data Availability

The original contributions presented in the study are included in the article, further inquiries can be directed to the corresponding author.
